# A foundation for reference models for drug combinations with an application to Loewe’s reference model

**DOI:** 10.1186/s12859-020-03771-4

**Published:** 2020-10-15

**Authors:** Wim De Mulder, Martin Kuiper

**Affiliations:** Department of Biology, NTNU, Realfagbygget, Trondheim, Norway

**Keywords:** Loewe, Complementary dose, Drug combinations, Equivalent dose, Reference model

## Abstract

**Background:**

Treating patients with combinations of drugs that have synergistic effects has become widespread practice in the clinic. Drugs work synergistically when the observed effect of a drug combination is larger than the effect predicted by the reference model. The reference model is a theoretical null model that returns the combined effect of given doses of drugs under the assumption that these drugs do not interact. There is ongoing debate on what it means for drugs to not interact. The controversy transcends mathematical punctuality, as different non-interaction principles result in different reference models. A famous reference model that has been in existence for already a long time is Loewe’s reference model. Loewe’s vision on non-interaction was purely intuitive: two drugs do not interact if all combinations of doses that result in a certain given effect lie on a straight line.

**Results:**

We show that Loewe’s reference model can be obtained from much more fundamental principles. First, we introduce the new notion of complementary dose. Secondly, we reformulate the existing concept of equivalent dose, whereby our formulation is more general than existing ones. Finally, a very general non-interaction principle is put forward. The proposed non-interaction principle represents a certain interplay between complementary and equivalent doses: drugs are non-interacting if complementarity is preserved under equivalence. It is then shown that Loewe’s reference model naturally follows from these principles by an appropriate choice of complementarity.

**Conclusions:**

The presented work increases insight into Loewe’s reference model for drug combinations, which is realized by the introduction of a very general non-interaction principle that does not refer to any specific dose-response curve, nor to any property of applicable dose-response curves.

## Background

For many diseases it has become standard practice to give drug combinations to patients. The biochemical rationale is that an effective drug combination may target multiple proteins or pathways, compared to mono-therapies [1]. The effects resulting from a drug combination may even be larger than what would have been expected based on the properties of the dose-response curves of the single drugs, in which case the effects are called synergistic. Diseases for which one has discovered drug combinations with synergistic effects include cancer [2], Parkinson’s disease [3], HIV [4–6] and asthma [7–10]. The impact of multi-therapies on such severe diseases explains why combinatorial drug screening and synergy scoring are very hot research topics [11]. A rigorous assessment of synergy in drug combinations must be based on the comparison of the observed effect of the drug combination with a non-interactive reference model [12]. The reference model is a theoretical model that produces the effect of a combination of given drugs under the assumption that these drugs do not interact. There is still wide debate on the precise meaning of non-interaction between drugs. Several researchers have put forward different non-interaction principles, resulting in dissimilar reference models. A very well established non-interaction principle is the one introduced by Bliss who associates a random variable with each drug, thereby assimilating non-interaction between the drugs with statistical independence between the corresponding random variables [13]. His reference model is still used to detect drug synergies, e.g. in the web tool SynergyFinder [14]. Another very popular reference model, which is also implemented in the aforementioned web tool as well as in CImbinator [15], is the Loewe additivity reference model [16]. Since the work in this paper relates to Loewe’s reference model, we provide a brief background of this model in section "Related work: Loewe’s reference model".

## Methods

In this paper we introduce the new concept of *complementary dose* (section "Complementary dose"). Two doses of the same drug are complementary with respect to a given effect if their sum produces that effect. The concept of *equivalent dose* is frequently used in the literature on drug combinations; it has been introduced to establish a certain similarity between doses of different drugs. In section "Equivalent dose" we introduce a general formulation of this notion. The formulation is radically different than existing ones: complementarity is associated with the linear transformation of basis vectors in two-dimensional Euclidean space. We also give a concrete example of such a linear transformation, to which we refer as ’the special case’. In section "Non-interaction principle" a new and very general non-interaction principle is introduced. It is stated in terms of a certain interplay between complementarity and equivalent doses. Having constructed this non-interaction principle, the definition of a general reference model then naturally follows (section "Construction of reference model"). The reference model for the special case of equivalent doses turns out to be Loewe’s reference model. Thus the main result of the paper is that a fundamental and general non-interaction principles is introduced, based on constructed notions of complementary and equivalent doses, and that Loewe’s reference model results as one member of the class of reference models that rely on this non-interaction principle.

## Notational conventions and assumptions

We consider two drugs, with corresponding dose-response curves $f_1$ and $f_2$. The dose-response curves are assumed to be *given*. An arbitrary dose of the *i*th drug is denoted by $u_i$ with $i \in \{1,2\}$. If another dose of the same drug is needed in the description, we denote this dose by $u'_i$. An arbitrary effect is denoted by *y*. If any of the two drugs is considered, we refer to this drug as the *i*th drug, while the other drug is then referred to as the *j*th drug.

We make the assumption that the dose-response curves are invertible. This assumption is common in the drug combinations literature, although it is often not explicitly stated, e.g. [17]. For simplicity, we assume that all doses are nonnegative, although this restriction can be dropped without altering any of the results below.

The effect produced by the reference model, which is constructed below, for given doses $u_1$ and $u_2$ will be denoted by ${\mathcal {F}}(u_1,u_2)$.

## Related work: Loewe’s reference model

Loewe states that two drugs are non-interacting if all combinations of doses that have a certain effect *y* lie on a straight line. Given an effect *y* and the doses $u^{\star }_1$ and $u^{\star }_2$ for which it holds that $f_1(u^{\star }_1)=f_2(u^{\star }_2)=y$, it is then the straight line from $(u^{\star }_1,0)$ to $(0,u^{\star }_2)$ that contains the pairs of doses $(u_1,u_2)$ that result in the effect *y* under the assumption of non-interaction. This straight line is given by the equation

$$\begin{aligned} \frac{u_1}{u^{\star }_1} + \frac{u_2}{u^{\star }_2} &= 1 \end{aligned}$$

Taking into account that $f_i(u^{\star }_i)=y$ or, equivalently, that $u^{\star }_i=f^{-1}_i(y)$, we find that Loewe’s reference model, denoted by ${\mathcal {F}}_L$, is given by the following definition.

### Definition 1

(*Loewe’s reference model*) The combined effect produced by Loewe’s reference model is given by $y={\mathcal {F}}_L(u_1,u_2)$ with *y* the solution of

1 $$\begin{aligned} \frac{u_1}{f^{-1}_1(y)} + \frac{u_2}{f^{-1}_2(y)} &= 1 \end{aligned}$$

## Complementary dose

We will introduce two concepts, namely complementary dose and equivalent dose. Both concepts apply to a given dose of a given drug. However, whereas the complementary dose returns a dose of the *same* drug, the equivalent dose returns a dose of *another* drug. The concept of complementary dose is introduced in this section, while the idea of equivalent dose is considered in the next section.

### Definition 2

(*Complementary dose*) Given an effect *y* and a dose $u_i$ with $0 \le u_i \le f^{-1}_i(y)$, we define $h_{i,y}$ as

2 $$\begin{aligned} h_{i,y}(u_i) &= -u_i + f^{-1}_i(y) \end{aligned}$$

The dose $h_{i,y}(u_i)$ is called the *complementary dose* of $u_i$ with respect to *y* or, in short, the complement of $u_i$ with respect to *y*.

The meaning of the definition is that if $u_i$ is a dose for which $0 \le u_i \le f^{-1}_i(y)$, and $u'_i$ is its complement with respect to *y*, then $f_i(u_i+u'_i)=y$. Thus two doses are complementary with respect to *y* if added together they produce the effect *y*.

The following observations can be made from the given definition:

- $h_{i,y}$ is the straight line from $(0,f^{-1}_i(y))$ to $(f^{-1}_i(y),0)$.
- The function $h_{i,y}$ is an affine transformation.

Since $h_{i,y}$ is an affine transformation, it can be described by a linear transformation. This requires to represent doses in two-dimensional Euclidean space, which can be performed by denoting a dose $u_i$ as the two-dimensional vector $U_i = (u_i,1)^T$.

The function $h_{i,y}(u_i)$ can then be represented as the matrix $H_{i,y}$:

3 $$\begin{aligned} H_{i,y} &= \begin{bmatrix} -1 &{} f^{-1}_i(y) \\ 0&{} 1 \end{bmatrix} \end{aligned}$$

Indeed, we have that

$$\begin{aligned} H_{i,y} \, U_i&= \begin{bmatrix} -1 &{} f^{-1}_i(y) \\ 0&{} 1 \end{bmatrix} \begin{bmatrix} u_i \\ 1 \end{bmatrix} \\= & {} \begin{bmatrix} -u_i + f^{-1}_i(y) \\ 1 \end{bmatrix} \\= & {} \begin{bmatrix} h_{i,y}(u_i) \\ 1 \end{bmatrix} \end{aligned}$$

## Equivalent dose

### Related work

The concept of equivalent dose is used in several papers on drug combinations. The idea is that a dose of a certain drug is equivalent to a dose of another drug if these doses show some similarity. An intuitive definition of equivalent dose was introduced by Lederer et al. [17]: two doses $u_i$ and $u_j$ are equivalent if these doses result in the same effect, i.e. if $f_i(u_i)=f_j(u_j)$. We use the notation $\xi _{ij}(u_j)$ to denote the dose that is equivalent to $u_j$. This means that according to the definition by Lederer et al., the dose $u_i$ of the *i*th drug that is equivalent to dose $u_j$ of the *j*th drug is given by

4 $$\begin{aligned} \xi _{ij}(u_j) &= f^{-1}_i(f_j(u_j)) \end{aligned}$$

The main reason that the concept of equivalent dose is used in the literature is to express the reference model in terms of a fixed drug, say the *i*th drug. This is done by transforming the given dose of the *j*th drug into its equivalent dose of the *i*th drug, and then expressing the combined effect produced by the reference model as the effect produced by the *i*th dose-response curve for the sum of $u_i$ and the equivalent dose of $u_j$. This means that one way to define a reference model is as

5 $$\begin{aligned} {\mathcal {F}}(u_1,u_2) &= f_i\Bigl (u_i + \xi _{ij}(u_j) \Bigr ) \end{aligned}$$

### A new general formulation

We introduce a new formulation of the concept of equivalent dose. Our formulation deviates from existing notions of equivalent dose in two ways:

- The dose that is equivalent to a given dose depends on a given effect. The reason is that below we will consider a certain interplay between complementary dose and equivalent dose, which requires that the equivalent dose depends on a given effect, just as the complementary dose depends on such a given effect (cf. section "Complementary dose").
- The equivalent dose is interpreted as a change of basis in two-dimensional Euclidean space.

This means that the transformation from a given dose to its equivalent dose can be represented by a general matrix

6 $$\begin{aligned} E_{ij,y} &= \begin{bmatrix} a_{ij,y} &{} b_{ij,y} \\ c_{ij,y} &{} d_{ij,y} \end{bmatrix} \end{aligned}$$

with $(a_{ij,y},c_{ij,y})^T$ and $(b_{ij,y},d_{ij,y})^T$ representing, in linear algebra terms, the new basis vectors, i.e. the transformations of the old basis vectors $(1,0)^T$ and $(0,1)^T$. We thus envisage the concept of equivalent dose as a change-of-basis linear transformation in two-dimensional space.

Depending on the intended precise interpretation of equivalent dose, the components of $E_{ij,y}$ may differ. Other values for these components imply other definitions of equivalent dose. It will be shown that Loewe’s reference model actually relies on a very simple change-of-basis transformation. We describe this transformation in the next subsection, while the use of it in the context of Loewe’s model is considered later.

### A special case

The simplest transformation scales both units vectors by a constant $\alpha _{ij,y}$, i.e.

7 $$\begin{aligned} E_{ij,y} &= \alpha _{ij,y} \, I \end{aligned}$$

with *I* denoting the identity matrix. Now, it is desired that for a given *y* and a given dose $u_j$ for which $f_j(u_j)=y$ it holds that the equivalent dose with respect to *y* is given by $u_i$ for which $f_i(u_i) = y$. In other words, that

$$\begin{aligned} E_{ij,y} \, (u_j,1)^T&= (f^{-1}_i(y),c)^T \end{aligned}$$

where *c* is an unimportant constant. By virtue of (7), and taking into account that $u_j=f^{-1}_j(y)$, this means that

$$\begin{aligned} \alpha _{ij,y} \,u_j &= f^{-1}_i(y) \\ \Leftrightarrow \alpha _{ij,y} \,f^{-1}_j(y) &= f^{-1}_i(y) \\ \Leftrightarrow \ \ \ \ \ \ \ \ \ \, \alpha _{ij,y} &= \frac{f^{-1}_i(y)}{f^{-1}_j(y)} \end{aligned}$$

Combining this with (7) this implies that

8 $$\begin{aligned} E_{ij,y} &= \frac{f^{-1}_i(y)}{f^{-1}_j(y)} \, I \end{aligned}$$

Further reference to $E_{ij,y}$ will denote this specific matrix, and not the general matrix given by (6), unless otherwise stated.

## Non-interaction principle

In this section we introduce a new non-interaction principle. The non-interaction principle will rely on the introduced notions of complementary and equivalent dose. We first describe, in section "General case", the principle for two drugs in the general case, i.e. for *any* matrix $E_{ij,y}$. The non-interaction principle is then specifically considered for the special case where $E_{ij,y}$ is given by (8) (cf. section "Special case" below).

### General case

Let us consider an effect *y* and a dose $0 \le u_j \le f^{-1}_j(y)$ of the *j*th drug. Consider its complementary dose which is, according to Definition 2, and taking into account (3), given by $U'_j = H_{j,y} \, U_j$, where $U_j=(u_j,1)^T$. Now consider the dose of the *i*th drug that is equivalent to $U'_j$, given by

9 $$\begin{aligned} U'_i &= E_{ij,y} \, U'_j \ = \ E_{ij,y} \, H_{j,y} \, U_j \end{aligned}$$

On the other hand, the equivalent dose to $U_j$ is given by $U_i = E_{ij,y} \, U_j$.

The non-interaction principle that we put forward then requires that $U_i$ and $U'_i$ are complementary doses, i.e. that it holds that

10 $$\begin{aligned} H_{i,y} \, U_i &= U'_i \end{aligned}$$

Simply put: *Non-interaction means that complementarity is preserved under equivalence*. Indeed, we have assumed that $U'_j$ and $U_j$ are complementary doses, i.e. $U'_j = H_{j,y} \, U_j$. Furthermore, the equivalent dose to $U'_j$ is $U'_i = E_{ij,y} \, U'_j$, and the equivalent dose to $U_j$ is $U_i = E_{ij,y} \, U_j$. Saying that the complementarity between $U'_j$ and $U_j$ also applies to the corresponding equivalent doses $U'_i$ and $U_i$ is indeed what is expressed by (10).

**Fig. 1:**
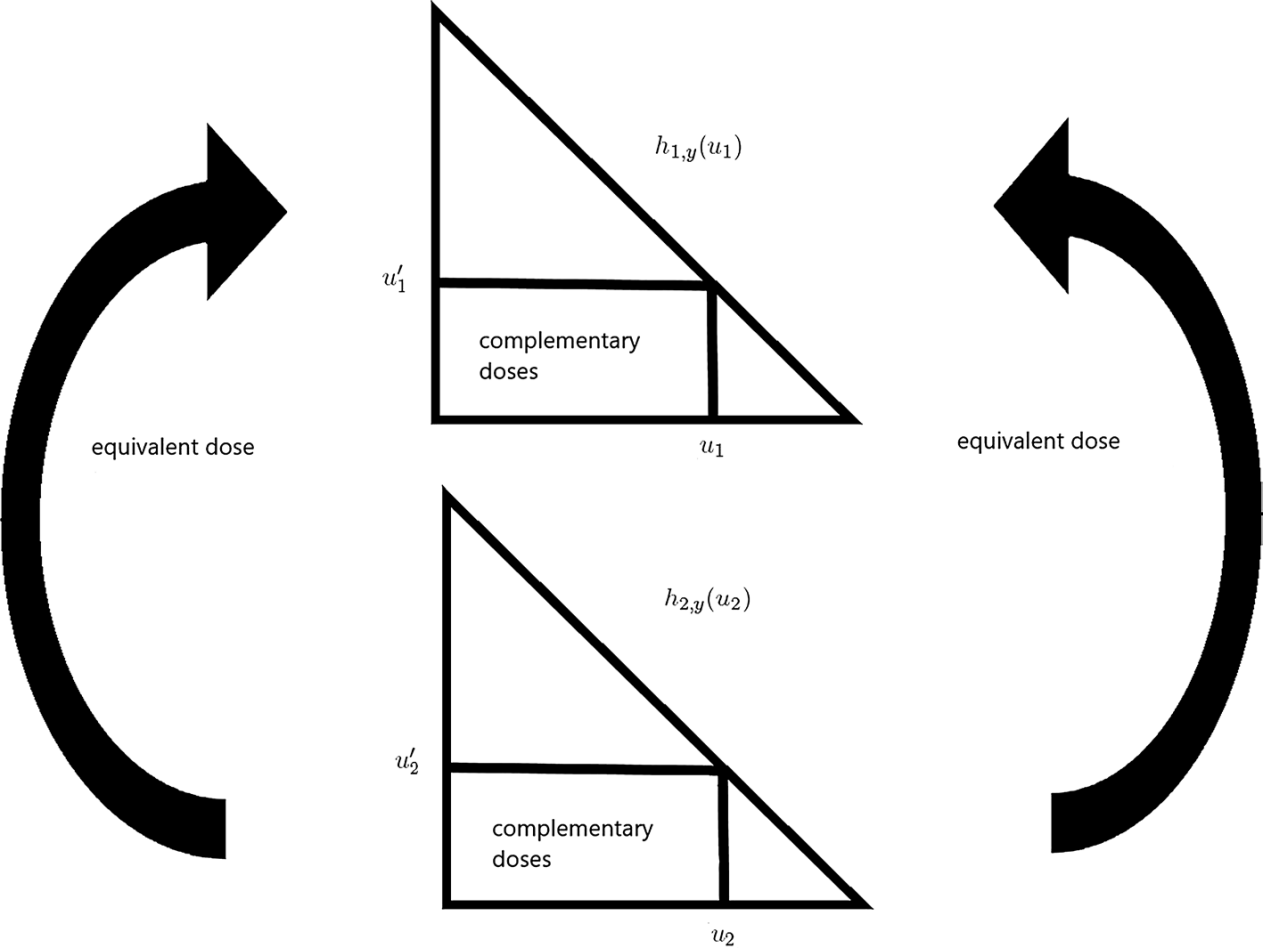
Illustration of the introduced non-interaction principle for two drugs

The principle is illustrated in Fig. 1. We summarize this principle in the following definition.

#### Definition 3

(*General non-interaction principle*) Two drugs are non-interacting if complementary doses are preserved under equivalence. This means that

11 $$\begin{aligned} H_{i,y}\, E_{ij,y} &= E_{ij,y}\, H_{j,y} \end{aligned}$$

for any effect *y*.

Notice that (11) is indeed the same as (10), by taking into account (9) and the fact that $U_i = E_{ij,y} \, U_j$. Equation (11) clearly shows that the non-interaction principle can also be interpreted as stating that the order of taking equivalent and complementary doses should not matter.

### Special case

For the special case (cf. section "A special case"), it follows from (3) and (8) that the non-interaction principle (11) is given by

$$\begin{aligned} \begin{bmatrix} -1 &{} f^{-1}_i(y) \\ 0&{} 1 \end{bmatrix} \frac{f^{-1}_i(y)}{f^{-1}_j(y)} \, I= & {} \frac{f^{-1}_i(y)}{f^{-1}_j(y)} \, I \begin{bmatrix} -1 &{} f^{-1}_j(y) \\ 0&{} 1 \end{bmatrix} \end{aligned}$$

It follows that this basic case results in a very severe restriction on the dose-response curves, which is summarized in the following definition.

#### Definition 4

(*Non-interaction principle for the special case*) Two drugs are non-interacting for the choice of *E* given by (8) if

12 $$\begin{aligned} f^{-1}_i(y) &= f^{-1}_j(y) \end{aligned}$$

## Construction of reference model

Having established the non-interaction principle, we introduce a general reference model in section "General case". This is then specialized to the case where the matrix *E* is given by (8).

### General case

Given are a dose $u_i$ of the *i*th drug and a dose $u_j$ of the *j*th drug. The question of constructing a reference model then amounts to describing the effect of combining doses $u_i$ and $u_j$ assuming that the two drugs obey the above described non-interaction principle. It is tempting to use the same formulation as for the explicit reference models by Lederer et al., cf. (5), where we simply replace their definition of equivalent dose by the one that we have introduced. This would result in:

13 $$\begin{aligned} {\mathcal {F}}_i(u_i,u_j) &= f_i\Bigl (u_i + \xi _{ij,y}(u_j) \Bigr ) \end{aligned}$$

where $i=1$ and $j=2$ or vice versa. For consistency reasons the choice should not matter, although this issue is not considered here. What is of interest here is that (13) does not describe a well-defined reference model as *y* is unknown, implying that $\xi _{ij,y}(u_j)$ is undetermined.

To circumvent this matter, let us start from the effect *y*, whatever its value is exactly, and consider ${\mathcal {F}}_i$, given by (13). The reference model ${\mathcal {F}}_i$ can only produce the effect *y* by combining the doses $u_i$ and $u_j$ if the equivalent dose to $u_j$ is complementary to $u_i$. That is, when $u_i = h_{i,y}\, \xi _{ij,y}\, u_j$. Indeed, it is only when this equality is true that it follows that the right hand side of (13) is given by

$$\begin{aligned} f_i\Bigl (u_i + \xi _{ij,y}(u_j) \Bigr) &= f_i\Bigl (u_i + h^{-1}_{i,y}(u_j)\Bigr ) \\ &= f_i\Bigl (u_i + h_{i,y}(u_i)\Bigr ) \\ &= y \end{aligned}$$

using Definition 2 of complementary doses.

In other words, we have found that *y* is determined by the equation $u_i = h_{i,y}\, \xi _{ij,y} \, u_j$. The reference model ${\mathcal {F}}_i(u_i,u_j)$ is therefore described as follows:

14 $$\begin{aligned} {\mathcal {F}}_i(u_i,u_j) &= y \end{aligned}$$

with *y* the solution of

15 $$\begin{aligned} h_{i,y} \, \xi _{ij,y} \, u_j &= u_i \end{aligned}$$

or in vector-matrix notation:

16 $$\begin{aligned} H_{i,y} \, E_{ij,y} \, U_j &= U_i \end{aligned}$$

### Special case

For the special case, *y* is, of course, also the solution of

17 $$\begin{aligned} H_{i,y} \, E_{ij,y} \, U_j &= U_i \end{aligned}$$

but with $E_{ij,y}$ given by (8). This means that the above equation reduces to

18 $$\begin{aligned} \begin{bmatrix} -1 &{} f^{-1}_i(y) \\ 0&{} 1 \end{bmatrix} \, \frac{f^{-1}_i(y)}{f^{-1}_j(y)} \, U_j &= U_i \end{aligned}$$

This results in two equations, but only the first one is of interest, since the second one gives the trivial identity $1=1$. From (18) it follows that the first equation is given by

$$\begin{aligned} - \frac{f^{-1}_i(y)}{f^{-1}_j(y)} \, u_j + \frac{\Bigl (f^{-1}_i(y)\Bigr )^2}{f^{-1}_j(y)} &= u_i \\ \Leftrightarrow \ \ \ \ \ - \frac{u_j}{f^{-1}_j(y)} + \frac{f^{-1}_i(y)}{f^{-1}_j(y)} &= \frac{u_i}{f^{-1}_i(y)} \end{aligned}$$

Applying the non-interaction principle for the special case, given by (12), the above equation turns out to be

$$\begin{aligned} - \frac{u_j}{f^{-1}_j(y)} + 1 &= \frac{u_i}{f^{-1}_i(y)} \\ \Leftrightarrow \frac{u_i}{f^{-1}_i(y)} + \frac{u_j}{f^{-1}_j(y)} &= 1 \\ \Leftrightarrow \frac{u_1}{f^{-1}_1(y)} + \frac{u_2}{f^{-1}_2(y)} &= 1 \end{aligned}$$

In other words, we have inferred Loewe’s reference model, as the above equation is the same as (1).

## Discussion

### Summary of our work

Reference models rely on a non-interaction principle. It is generally thought that different reference models are distinguished by different non-interaction principles. In this paper we have shown that it is possible to construct a general non-interaction principle that may be accepted to apply to all non-reference models. The concept that may differ from reference model to reference model is the formulation of equivalent dose. Indeed, we have demonstrated that Loewe’s reference model follows from a specific instantiation of our general formulation of equivalent dose: equation (8) is a specific example of an equivalent dose definition that obeys the general formulation given by (6). In contrast, the general non-interaction principle that we introduced, given by Definition 3, does not require any additional specification: it applies to Loewe’s reference model, just as it may apply to any other reference model. Of course, because the non-interaction principle is described in terms of a certain interplay between complementary dose and equivalent dose, the specific non-interaction principle in the case of Loewe shall be different than for other reference models (which rely on a different instantiation of equivalent dose).

### Novelties in the paper

One novelty of our work is that the introduced non-interaction principle (cf. Definition 3) is very general: it does not refer to any specific dose-response curve, nor to any property of applicable dose-response curves. It just applies to all dose-response curves, provided they are invertible (because of the presence of the matrix *H* that relies on the inverse of the involved dose-response curve).

Another novelty is that the non-interaction principle recognizes that the concept of equivalent dose, which is often employed in the literature in the construction of a non-interaction principle, is not sufficient to define non-interaction. An additional notion is needed, namely complementary dose. Non-interaction can then be defined in terms of a certain interplay between equivalence and complementarity. More specifically: drugs are non-interacting if complementarity is preserved under equivalence.

From the introduced non-interaction principle, which relies on the constructed notions of equivalence and complementarity, we were able to naturally derive Loewe’s reference model. That is, Loewe’s reference model is a *consequence* of the introduced non-interaction principle. In contrast to Loewe, we did not have to assume from the outset that non-interaction means that the reference model is a set of straight lines (one straight line for each given effect). In other words: the principles we introduced are more fundamental than the assumptions that Loewe made. The fact that one possible interpretation of non-interaction corresponds to straight lines does not have to be taken for granted, it simply follows from the introduced non-interaction principle for a certain, simple choice of equivalent dose.

### Future work

The non-interaction principle we have introduced is very general, but it is still formulated in the rather restrictive framework of linear algebra. In particular, the concepts of complementary and equivalent doses are defined in terms of linear transformations. The class of reference models would be extended by allowing complementarity and equivalence to be defined in terms of nonlinear transformations. For example, the often used definition of equivalence as given by (4) is nonlinear.

Furthermore, it would be useful work to consider if other well known reference models could be deduced from the established framework.

## Data Availability

Not applicable.
